# Probing the Effects of N-Acetylglucosamine and Diazepam Combination on Oxidative Stress and Epileptogenesis-Associated Genes in Murine Brain

**DOI:** 10.3390/cimb48040385

**Published:** 2026-04-09

**Authors:** Abigail M. Akhigbemen, Justice Osemede, Elohor E. Okpakpor, David C. Orji, Israel O. Bolanle, Raymond I. Ozolua

**Affiliations:** 1 Department of Pharmacology & Toxicology, Faculty of Pharmacy, University of Benin, Benin-City 300001, Nigeria; abigail.omo-isibor@uniben.edu (A.M.A.); osemedejustice@gmail.com (J.O.); davidorji64@gmail.com (D.C.O.); ozolua@uniben.edu (R.I.O.); 2 Department of Science Laboratory Technology, Faculty of Life Sciences, University of Benin, Benin-City 300001, Nigeria; elohor.okpakpor@uniben.edu; 3 Centre for Biomedicine, Hull York Medical School, University of Hull, Hull HU6 7RX, UK

**Keywords:** epilepsy, inflammation, antioxidant, N-acetyl glucosamine, gene expression

## Abstract

A body of evidence suggests that upregulating *O*-GlcNAcylation, a reversible post-translational modification of serine and threonine residues on target proteins, is beneficial in neurological diseases. However, this phenomenon is currently underexplored in the pharmacotherapy of epilepsy. Therefore, we aimed to explore the potential effects of combining N-acetylglucosamine (GlcNAc), a precursor for *O*-GlcNAcylation, and a centrally acting benzodiazepine (diazepam) on oxidative stress, a known driver of epilepsy, and some epileptogenesis-associated genes. Mice (*n* = 10) were randomly assigned to treatment groups and treated with varied oral doses (100, 200, and 400 mg/kg) of GlcNAc in combination with diazepam (1 mg/kg) for 14 days. Following this, seizure was chemically induced with 70 mg/kg pentylenetetrazol intraperitoneally. Brains of treated mice were excised for antioxidant assays and to determine the expression of genes associated with epileptogenesis: potassium chloride co-transporter (*KCC4*), interleukin (*IL-6*), tumour necrosis factor-α (*TNF-α*), and brain-derived neurotrophic factor (*BDNF*). Our findings suggest that GlcNAc, when concurrently administered with diazepam, prevents oxidative stress and reduces the gene expression of *IL-6*, a cytokine associated with neuroinflammation and seizures, whilst increasing the gene expression of *KCC4*, an ion co-transporter that promotes antiepileptogenesis.

## 1. Introduction

Epilepsy, a brain disorder characterised by the constant and unpredictable episodes of seizures [1], is the second most prevalent neurological condition in the world, affecting over 70 million people, and 80% of this number live in developing countries [2]. Epilepsy is strongly associated with impaired quality of life, much more than is seen in many other chronic illnesses [3]. Despite this, not much attention is given to the quality of life of people with epilepsy, other than targeting symptom reduction [4]. People with epilepsy are often stigmatised with reduced life opportunities in every aspect which impedes their quality of life [5]. The underlying causes of seizures vary and may include genetic predispositions, structural brain abnormalities, or metabolic disturbances, all of which can result in abnormal electrical activity that disrupts normal brain function [6].

Currently used anti-seizure agents (predominantly GABAmimetics) typically inhibit neurotransmission, which results in adverse effects such as sedation, cognitive deficits, mood distortion, weight gain, as well as teratogenicity [7]. Another drawback of these agents is that, despite their effectiveness, refractoriness has been observed in some patients [8]. These adverse effects often result in poor patient compliance and attendant reduced effectiveness. Also, some antiepileptic drugs increase seizure frequency and severity, and change seizure type [9]. These disadvantages may result in depression and suicidal attempts and increase the burden of this disease on caregivers and society at large, which makes the search for better drugs a continuous exercise.

Oxidative stress in the brain is a major contributing factor to the onset and progression of epilepsy [10]. Therefore, reducing oxidative stress could be a viable strategy for treating epilepsy. Furthermore, a body of evidence suggests that upregulating *O*-GlcNAcylation could be beneficial in the treatment of neurological disorders [11]. *O*-GlcNAcylation, a dynamic, reversible post-translational modification of proteins that involves the attachment of GlcNAc residues to serine or threonine residues on target proteins [12,13], affects several cellular functions, such as cytoskeletal organisation, metabolism, inflammation, trafficking, protein modification, and signalling pathways [14,15]. However, this phenomenon is yet to be fully explored as a treatment strategy in the management of epilepsy.

In humans suffering from epilepsy and in several rodent epilepsy models, glucose metabolism is impaired. This deficiency in glucose and energy has been linked to the generation of seizures, since the stabilisation of membrane action potentials and regulated neural signalling requires high amounts of energy. Stewart et al. [16] showed that an acute increase in protein *O*-GlcNAcylation limits epileptiform activity in the hippocampus. Therefore, in this study, we have evaluated the combined effect of N-acetylglucosamine (GlcNAc), a monosaccharide derivative of glucose and precursor of *O*-GlcNAcylation, and diazepam, the benzodiazepine of choice used for the management of acute seizures [17], in an animal model of epilepsy. Specifically, we have examined the effect of the combination of these drugs on oxidative stress biomarkers and the expression of some genes implicated in the pathology and progression of epilepsy, namely, potassium chloride co-transporter-4 (*KCC4*), interleukin-6 (*IL-6*), tumour necrosis factor -α (*TNF-α*), and brain-derived neurotrophic factor (*BDNF*) [18,19,20].

## 2. Materials and Methods

### 2.1. Animals

Adult male Swiss albino mice (weighing between 20 and 30 g) were assigned to five groups (*n* = 10). Animals in group 1 received 0.2 mL/day of distilled water (control), while those in groups II, III, and IV received trial-optimised oral doses of 100, 200, and 400 mg/kg/day GlcNAc, respectively, concurrently with 1 mg/kg diazepam intraperitoneally. Animals in group V received 1 mg/kg/day diazepam intraperitoneally (standard). Treatment lasted 14 consecutive days. An hour post-treatment on the 14th day, 70 mg/kg pentylenetetrazol (PTZ) was administered intraperitoneally to all groups [21]. Thereafter, the animals were humanely sacrificed, whole brains were harvested from some mice (*n* = 6) and homogenised for antioxidant assay, while the hippocampus and cortex of others (*n* = 4) were stored in Trizol reagent for the determination of the expression of epileptogenesis-associated genes. Animals were handled in accordance with international protocols for the use of animals in experiments [22], and the adopted protocols met the requirements of the ARRIVE 2.0 guidelines (https://arriveguidelines.org/arrive-guidelines/sample-size; accessed 10 January 2026).

### 2.2. Assay of Brain Antioxidants

The homogenised brain samples were assayed for glutathione peroxidase (GPx) [23], superoxide dismutase (SOD) [24], catalase (CAT) [25], and malondialdehyde (MDA) [26]. Briefly, GPx catalyses the reduction of hydrogen peroxide in the presence of reduced glutathione, and the absorbance of the remaining reduced glutathione was measured after its reaction with Ellman’s reagent at 412 nm [23]. SOD was measured by determining the absorbance of the auto-oxidation of adrenochrome derived from adrenaline at 420 nm [24]. CAT was assayed by measuring the activity of catalase in decomposing hydrogen peroxide to water and oxygen in the presence of potassium permanganate. The absorbance was measured at 480 nm [25]. To determine MDA concentration, brain MDA, a by-product of lipid peroxidation, was reacted with thiobarbituric acid to form a pink-coloured MDA-TBA adduct, which was measured at 535 nm [26].

### 2.3. Isolation and Purification of Total RNA

Excised mice brains preserved in Trizol were homogenised manually with a pestle in Eppendorf tubes to expose cell nuclei, after which the brain tissues were partitioned using chloroform as a gradient separation medium. Isoamyl alcohol was then added, followed by DNase (NEB) treatment for 10 min; the RNA pellet was rinsed with alcohol to remove contaminants. The resulting DNase-free RNA was suspended in nuclease-free water, and purity was evaluated by measuring absorbance at 260 and 280 nm [27].

### 2.4. Complementary DNA (cDNA) Synthesis: Polymerase Chain Reaction (PCR) and Amplification of Genes of Interest

The total RNA obtained was converted to complementary DNA (cDNA) using the reverse transcriptase polymerase chain reaction (RT-PCR). The genes of interest were then amplified using a designed and optimised forward and reverse primers (Table 1). Glyceraldehyde-3-phosphate dehydrogenase (GAPDH) was employed as the housekeeping gene, while PCR Master Mix catalysed the amplification with the aid of a thermocycler (Eppendorf Mastercycler AG 22331, Barkhausenweg 1, 22339 Hamburg, Germany) for 30 cycles [28].

### 2.5. Agarose Gel Electrophoresis

Amplicons derived from RT-PCR products were subjected to electrophoresis (1% agarose gel) to facilitate the migration of the amplicons from the anode to the cathode at a constant voltage, following which the relative density and intensity of the gene bands were quantified using ImageJ 1.54k (September 2024) software.

### 2.6. Statistical Analysis

Data obtained are expressed as Mean ± S.E.M (standard error of mean) and analysed using one-way ANOVA followed by Tukey’s multiple comparison test (GraphPrism^®^ version 6, San Diego, CA, USA). *p* < 0.05 was considered significant.

## 3. Results

### 3.1. Evaluation of the Antioxidant Property of GlcNAc

Data shown in Table 2 indicate that GlcNAc did not cause any significant difference in the expression of GPx, MDA, and SOD in whole brain of treated mice when compared to the control and the group treated with only diazepam. However, doses of 100 and 200 mg/kg/day GlcNAc significantly increased the expression of the CAT (*p* < 0.05 and *p* < 0.01, respectively) when compared to the control.

### 3.2. Effect of GlcNAc on the Expression of Epileptogenic Cytokines in the Hippocampus and Cortex of Mice

#### 3.2.1. *KCC4*

**Figure 1:**
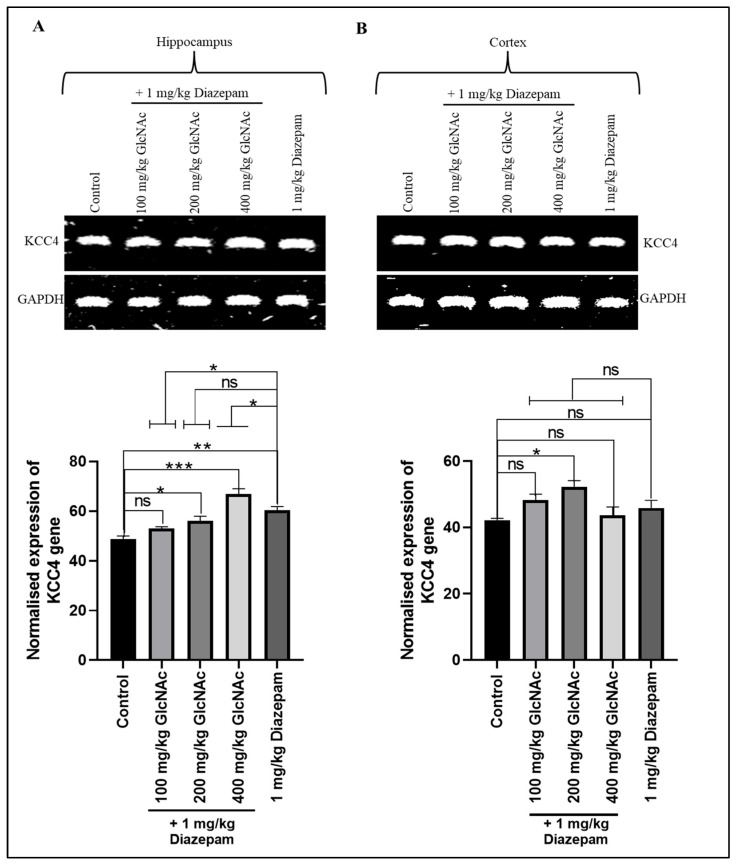
Expression of the *KCC4* gene in the (**A**) Hippocampus (**B**) Cortex of mice following concurrent treatment of mice with GlcNAc and diazepam for 14 days. (**A**) **Upper panel**: Representative KCC4 gene expression in the hippocampus following treatments. **Lower panel**: Densitometric analysis of *KCC4* gene expression in the hippocampus, normalised to GAPDH (loading control) (**B**) **Upper panel**: Representative *KCC4* gene expression in the cortex following treatments. **Lower panel**: Densitometric analysis of *KCC4* gene expression in the cortex, normalised to GAPDH (loading control). Normalised data are expressed as the mean ± SEM from *n* = 4. * *p* < 0.05, ** *p* < 0.01, *** *p* < 0.001, ‘ns’ for not significant. All data were compared with the control and the group treated with diazepam alone. *KCC4*: potassium chloride co-transporter, *GAPDH*: Glyceraldehyde 3-phosphate dehydrogenase, GlcNAc: N-acetylglucosamine.

There was a significant increase in *KCC4* gene expression in the hippocampus of mice that received 200 and 400 mg/kg/day GlcNAc concurrently with diazepam (*p* < 0.05, *p* < 0.0001, respectively), and it was also significantly (*p* < 0.05) expressed at the dose of 1 mg/kg/day diazepam compared to control (Figure 1A). Also, in the cortex, there was a significant (*p* < 0.05) increase in *KCC4* gene expression at the dose of 200 mg/kg/day GlcNAc when combined with diazepam (Figure 1B). When compared with diazepam only, in the hippocampus, there was a significant decrease (*p* < 0.05) and increase (*p* < 0.05) in *KCC4* gene expression following treatment with 100 mg/kg and 400 mg/kg, respectively, with diazepam (Figure 1A). On the other hand, in the cortex, there was no significant change in *KCC4* gene expression when mice were treated concurrently with GlcNAc and diazepam compared with diazepam-only treatment (Figure 1B).

#### 3.2.2. *IL-6*

**Figure 2:**
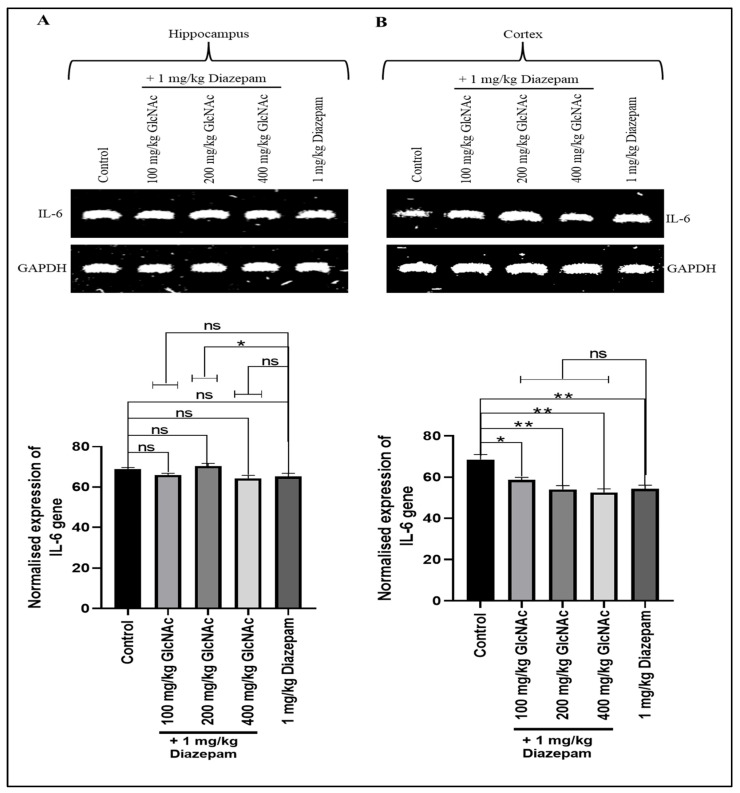
Expression of the *IL-6* gene in the (**A**) Hippocampus (**B**) Cortex of mice following concurrent treatment of mice with GlcNAc and diazepam for 14 days. (**A**) **Upper panel**: Representative *IL-6* gene expression in the hippocampus following treatments. **Lower panel**: Densitometric analysis of *IL-6* gene expression in the hippocampus, normalised to GAPDH (loading control) (**B**) **Upper panel**: Representative *IL-6* gene expression in the cortex following treatments. **Lower panel**: Densitometric analysis of *IL-6* gene expression in the cortex, normalised to GAPDH (loading control). Normalised data are expressed as the mean ± SEM from *n* = 4. * *p* < 0.05, ** *p* < 0.01, ‘ns’ for not significant. All data were compared with the control and the group treated with diazepam alone. *IL-6*: interleukin-6, *GAPDH*: Glyceraldehyde 3-phosphate dehydrogenase, GlcNAc: N-acetylglucosamine.

Figure 2A shows that when compared with the control, all doses of GlcNAc administered concurrently with diazepam did not significantly alter *IL-6* gene expression in the hippocampus of mice, but in the cortex (Figure 2B), the combination significantly decreased *IL-6* gene expression at doses of 100, 200, and 400 mg/kg/day (*p* < 0.05, *p* < 0.01). On the other hand, when compared with the group treated with diazepam only, concurrent treatment of diazepam with 200 mg/kg GlcNAc caused a significant increase in *IL-6* gene expression in the hippocampus (Figure 2A). However, there is no significant change in the expression of the *IL-6* gene in the cortex when compared with the group treated with diazepam only (Figure 2B).

#### 3.2.3. *TNF-α*

**Figure 3:**
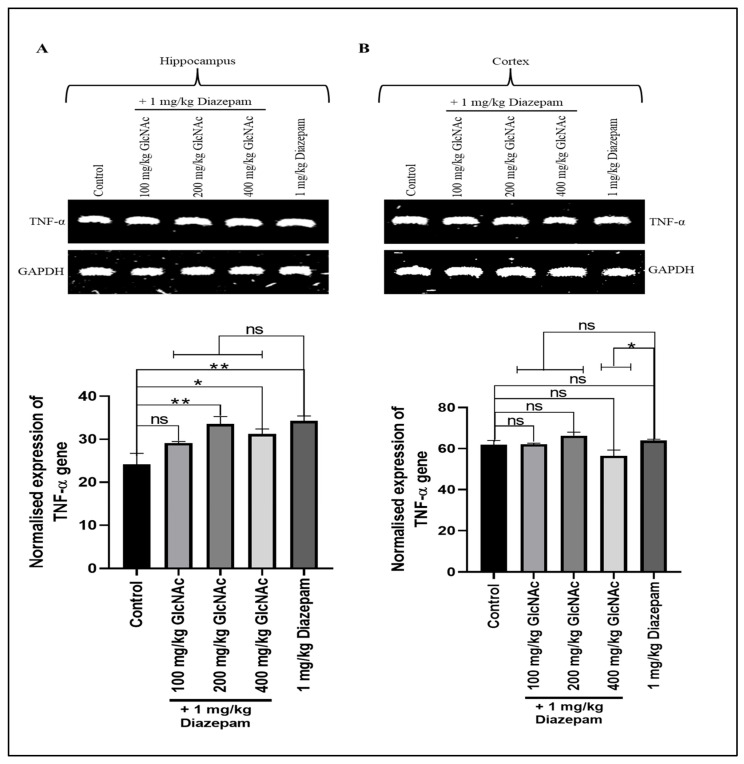
Expression of the *TNF-α* gene in the (**A**) Hippocampus (**B**) Cortex of mice treated concurrently with GlcNAc and diazepam for 14 days. (**A**) **Upper panel**: Representative *TNF-α* gene expression in the hippocampus following treatments. **Lower panel**: Densitometric analysis of *TNF-α* gene expression in the hippocampus, normalised to GAPDH (loading control) (**B**) **Upper panel**: Representative *TNF-α* gene expression in the cortex following treatments. **Lower panel**: Densitometric analysis of *TNF-α* gene expression in the cortex, normalised to GAPDH (loading control). Normalised data are expressed as the mean ± SEM from *n* = 4. * *p* < 0.05, ** *p* < 0.01, ‘ns’ for not significant. All data were compared with the control and the group treated with diazepam alone. *TNF-α*: tumour necrosis factor -α, *GAPDH*: Glyceraldehyde 3-phosphate dehydrogenase, GlcNAc: N-acetylglucosamine.

There was a significant increase in *TNF-α* gene expression in the hippocampus of mice that received 200 and 400 mg/kg/day GlcNAc concurrently with diazepam (*p* < 0.01, *p* < 0.05, respectively); the expression was also significant (*p* < 0.01) with 1 mg/kg/day diazepam compared to control (Figure 3A). However, in the cortex, there was no significant increase in *TNF-α* gene expression at the doses of GlcNAc concurrently administered with 1 mg/kg/day diazepam (Figure 3B). Also, in the hippocampus, when compared with diazepam-only, there was no significant change in *TNF-α* gene expression (Figure 3A). On the other hand, in the cortex, there was a significant decrease (*p* < 0.05) in *TNF-α* gene expression when mice were treated with 400 mg/kg/day GlcNAc and 1 mg/kg/day compared with the expression occurring with diazepam-only treatment (Figure 3B).

#### 3.2.4. *BDNF*

**Figure 4:**
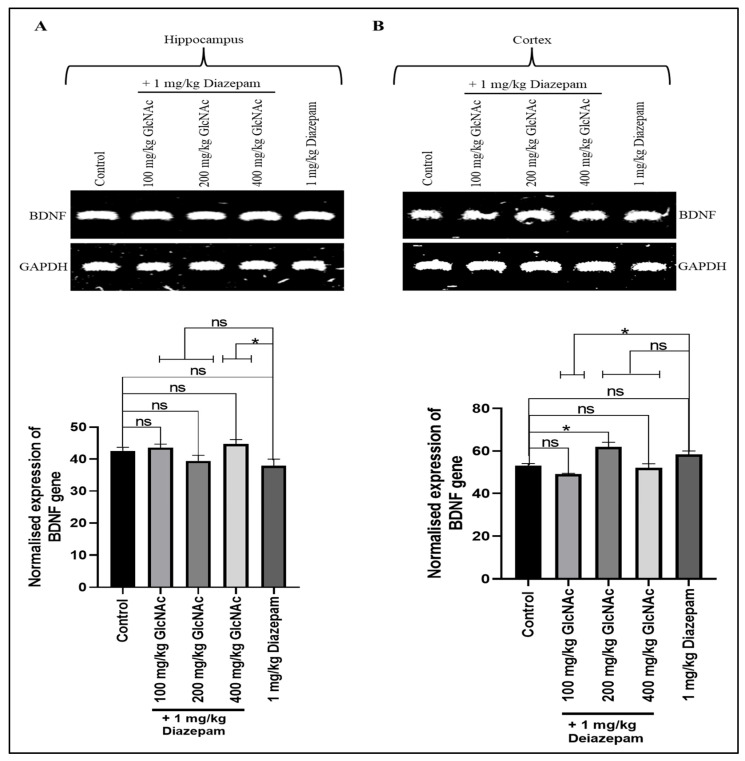
Expression of the *BDNF* gene in the (**A**) Hippocampus (**B**) Cortex of mice treated with GlcNAc and diazepam concurrently. (**A**) **Upper panel**: Representative *BDNF* gene expression in the hippocampus following treatments. **Lower panel**: Densitometric analysis of *BDNF* gene expression in the hippocampus, normalised to GAPDH (loading control) (**B**) **Upper panel**: Representative *BDNF* gene expression in the cortex following treatments. **Lower panel**: Densitometric analysis of *BDNF* gene expression in the cortex, normalised to GAPDH (loading control). Normalised data are expressed as the mean ± SEM from *n* = 4. * *p* < 0.05, ‘ns’ for not significant. All data were compared with the control and the group treated with diazepam alone. *BDNF*: brain-derived neurotrophic factor, *GAPDH*: Glyceraldehyde 3-phosphate dehydrogenase, GlcNAc: N-acetylglucosamine.

As shown in Figure 4A, when compared with the control, doses of GlcNAc administered concurrently with diazepam did not significantly alter *BDNF* gene expression in the hippocampus of mice, but in the cortex (Figure 4B), the combination significantly increased *BDNF* gene expression at 200 mg/kg/day (*p* < 0.05). On the other hand, when compared with the group treated with diazepam only, treatment with 400 mg/kg/day GlcNAc and 1 mg/kg/day diazepam caused a significant increase (*p* < 0.05) in *BDNF* gene expression in the hippocampus (Figure 4A). Also, there was a significant decrease (*p* < 0.05) in the expression of *BDNF* in the cortex at 100 mg/kg/day + 1 mg/kg/day when compared with the group treated with diazepam only (Figure 4B).

## 4. Discussion

In this study, we have examined the combined effect of GlcNAc, a precursor for O-GlcNAcylation, and an anticonvulsant benzodiazepine (diazepam) on oxidative stress biomarkers and the expression of some genes implicated in the pathology and progression of epilepsy in mice. We used PTZ to induce convulsions in mice [21]. Experimental data have suggested that oxidative stress may play a role in the pathophysiology of seizures, epilepsy, and epileptogenesis [10]. Thus, lowering oxidative stress may be an effective strategy to treat epilepsy since it reduces free radical-induced neuroinflammation [10]. Results from our study showed that catalase levels were elevated when 100 and 200 mg/kg GlcNAc were administered concurrently with diazepam for 14 days. Catalase, an enzyme ubiquitous in all living organisms, is essential for reducing oxidative stress because it catalyses the breakdown of hydrogen peroxide into oxygen and water. This prevents reactive oxygen species from building up and causing oxidative damage to tissues and cells [29]. The fact that when compared to control, there was a significant increase in catalase after concurrent treatment with GlcNAc and diazepam—but not diazepam alone—suggests that the combination of GlNAc and diazepam could mitigate oxidative stress-driven epilepsy.

Neuronal chloride homeostasis is crucial for sustaining cellular excitability and the proper functioning of neural circuits [30]. While regulation of intracellular chloride concentration is vital for effective GABAergic neurotransmission, the cation-chloride cotransporters (CCCs) have been adjudged indispensable in maintaining this homeostasis by mediating the movement of chloride ions across cell membranes [31]. The expression of potassium chloride co-transporters, a group of proteins that belong to the solute carrier family 12 (SLC12) of CCCs, is significantly downregulated by epileptogenic stimuli [32]. These transporters, particularly in the hippocampus, weaken GABA-mediated inhibition and promote hyper-excitability [33]. This leads to a cycle of seizure induction and propagation [33,34]. Impairment in KCCC function, often due to genetic mutations in the *SLC12a5* gene or altered expression, has been linked to various forms of epilepsy, including epilepsy of infancy with migrating focal seizures and Mesial Temporal Lobe epilepsy [35]. Our study showed a significant rise in *KCC4* expression in both the hippocampus and cortex of mice, suggesting that GlcNAc and diazepam reduce hyper-excitability. This observation suggests a potential strategy to limit epileptogenesis. However, further studies will be required to validate this.

Interleukins are a diverse group of cytokines involved in the regulation and mediation of inflammation and immune responses [36]. They can have both pro-inflammatory and anti-inflammatory effects [36]. Pro-inflammatory interleukins, particularly IL-1β, IL-4, and IL-6, play a crucial role in the inflammatory processes associated with epilepsy [37]. These cytokines contribute to neuronal hyperexcitability, blood–brain barrier disruption, and neurodegeneration, thereby enhancing seizure susceptibility and exacerbation. The interplay between pro-inflammatory cytokines and neuroinflammation creates a vicious cycle that exacerbates epilepsy [36]. In the pathophysiology of epilepsy, neuroinflammation plays a pivotal role in promoting epileptogenesis, with IL-6 emerging as a central inflammatory mediator [38]. IL-6 has also been shown to be the most consistently implicated cytokine in patients with epilepsy [39]. From our findings, *IL-6* gene expression in the hippocampus was not significantly affected by the concurrent administration of the two drugs. Meanwhile, in the cortex, there was a significant decrease in expression. This suggests that the combination may have anti-epileptogenic properties acting through anti-neuroinflammatory processes.

TNF-α plays a role in the pathophysiology of epilepsy by modulating neuroinflammation, astrocyte activity, and neuronal excitability [40]. Elevated levels of TNF-α have been implicated in seizure recurrence [41]. Cytokines contribute to seizure development through multiple mechanisms that promote neuronal hyper-excitability and disrupt the balance between excitation and inhibition in the brain [40]. Our study showed there was increased expression of *TNF-α* in the hippocampus following treatment with 200 and 400 mg/kg/day GlcNAc and 1 mg/kg/day diazepam. However, cortical levels of *TNF-α* were not significantly different from control. While it is currently unclear, the rise in *TNF-α* gene expression in the hippocampus could be because *TNF-α* is rapidly secreted as an immediate-early gene in response to diverse stimuli, including pathogenic infections, other cytokines, and environmental stressors, to ensure rapid and effective immune defence [42].

BDNF is critical for neuronal survival, differentiation, synaptic plasticity, and overall brain function [43]. In epilepsy, BDNF can have both pro-epileptogenic and anti-epileptogenic effects [44]. This dual function may arise from its role in regulating both glutamatergic and GABAergic transmission. It is suggested that BDNF reduces damage to the central nervous system caused by epilepsy through its neurotrophic effects, hence inhibiting epileptogenesis [45]. This is thought to occur via its binding to tropomyosin receptor kinase (TrkB) and neurotrophin receptor p75 receptors [45,46]. On the other hand, increased *BDNF* mRNA and TrkB receptor expression have consistently been observed in epileptogenic brain regions such as the hippocampus and amygdala, in both animal and human models [47]. In our study, hippocampal *BDNF* levels in mice treated with GlcNAc and diazepam were not significantly different from those in control mice. In the cortex, there was an increase at 200 mg/kg/day dose, which was not significant when compared to diazepam alone.

In summary, our findings suggest that when GlcNAc and diazepam are administered concurrently to mice, oxidative stress is prevented, the gene expression of *IL-6*, a cytokine linked to neuroinflammation and seizures, is decreased, and the gene expression of KCC4, an ion co-transporter that promotes antiepileptogenesis, is increased. These observations offer promising insights into the pharmacotherapy of epilepsy. However, we acknowledge some limitations to this study and recognise that more investigations will be needed to further elucidate these initial findings. For example, we determined gene expressions by conventional RT-PCR because it suffices as an applicable method to help achieve our objective. Meanwhile, qRT-PCR would have been more appropriate. Furthermore, we explored the hypothesis that continuous treatment with GlcNAc will favour OGT-mediated upregulation of *O*-GlcNAcylation, as supported by a body of literature [48]. However, measuring the *O*-GlcNAc levels, as well as the activities of the enzymes of *O*-GlcNAc cycling, *O*-GlcNAc transferase and *O*-GlcNAcase, will be useful and help support our conclusions. We aim to look at these gaps in future studies.

## 5. Conclusions

Our findings suggest that the concurrent administration of GlcNAc and diazepam prevents oxidative stress, increases the gene expression of the ion co-transporter, KCC4, that promotes antiepileptogenesis, whilst decreasing the expression of genes that are pro-neuroinflammatory and epileptogenic. These effects vary based on brain region, and further studies will be required to validate these observations.

## Figures and Tables

**Table 1:** The list of primers designed, optimised, and synthesised for each gene of interest.

Gene Name	Forward Primer	Reverse Primer
*IL-6*	GTCTGTAGCTCATTCTGCTCTG	GAAGGCAACTGGATGGAAGT
*TNF-α*	CTGAGTTCTGCAAAGGGAGAG	CCTCAGGGAAGAATCTGGAAAG
*BDNF*	TCCTAGAGAAAGTCCCGGTATC	GCAGCCTTCCTTGGTGTAA
*KCC4*	TGTACCACCTCAGGATCAGT	GTGACCTCTGCTCCATCATTAG

**Table 2:** Effect of concurrent daily administration (×14) of GlcNAc and diazepam on brain oxidative stress biomarkers of PTZ-treated mice.

	Glutathione Peroxidase (U/g Prot)	Superoxide Dismutase (U/g Prot)	Catalase (U/g Prot)	Malondialdehyde (µmol/g Prot)
Distilled water	4.13 ± 0.41	1.22 ± 0.0689	0.64 ± 0.035	1.26 ± 0.137
100 mg/kg GlcNAc + 1 mg/kg diazepam	3.62 ± 0.38	1.98 ± 0.120	1.04 ± 0.061 *	1.55 ± 0.212
200 mg/kg GlcNAc + 1 mg/kg diazepam	5.07 ± 0.78	2.48 ± 0.217	1.21 ± 0.125 **	0.84 ± 0.145
400 mg/kg GlcNAc + 1 mg/kg diazepam	5.94 ± 0.79	1.05 ± 0.055	0.56 ± 0.0253	2.00 ± 0.271
1 mg/kg diazepam	4.53 ± 0.56	1.54 ± 0.142	0.75 ± 0.065	1.42 ± 0.162

* *p* < 0.05, ** *p* < 0.01 versus control. All comparisons with the group treated with diazepam were not significant. *n* = 6 mice per group.

## Data Availability

The data presented in this study are available on request from the corresponding author. The data are not publicly available due to privacy or ethical restrictions.

## Abbreviations

BDNF	brain-derived neurotrophic factor
CAT	catalase
CCCs	cation-chloride cotransporters
DNA	deoxyribonucleic acid
GAPDH	glyceraldehyde-3-phosphate dehydrogenase
GlcNAc	N-acetylglucosamine
GPx	glutathione peroxidase
IL-6	interleukin-6
KCC4	potassium chloride co-transporter
MDA	malondialdehyde
PTZ	pentylenetetrazol
RNA	ribonucleic acid
RT-PCR	reverse Transcription Polymerase Chain Reaction
SLC12	solute carrier family 12
SOD	superoxide dismutase
TNF-α	tumour necrosis factor -α
TrkB	tropomyosin receptor kinase
